# Prognostic value of suPAR and hsCRP on acute kidney injury after cardiac surgery

**DOI:** 10.1186/s12882-021-02322-0

**Published:** 2021-04-07

**Authors:** Sebastian Roed Rasmussen, Rikke Vibeke Nielsen, Rasmus Møgelvang, Sisse Rye Ostrowski, Hanne Berg Ravn

**Affiliations:** 1 Department of Cardiothoracic Anaesthesiology, Rigshospitalet, Copenhagen University Hospital, Copenhagen, Denmark; 2 Department of Clinical Medicine, Faculty of Health and Medical Sciences, University of Copenhagen, Copenhagen, Denmark; 3 Clinical Cardiovascular Research Unit, University of Southern Denmark, Svendborg, Denmark; 4 Department of Clinical Immunology, Rigshospitalet, Copenhagen University Hospital, Copenhagen, Denmark; 5 Department of Anaesthesiology, Odense University Hospital, University of Southern Denmark, Odense, Denmark

**Keywords:** Biomarkers, Cardiac anaesthesia, Cardiac surgery, Risk prediction, Acute kidney injury

## Abstract

**Background:**

Acute kidney injury (AKI) represents a serious complication following cardiac surgery. Adverse outcome after cardiac surgery has been observed in the presence of elevated levels of soluble urokinase-type plasminogen activator receptor (suPAR) and high-sensitivity C-Reactive Protein (hsCRP). The aim of study was (i) to investigate the relationship between preoperative elevated levels of suPAR and hsCRP and postoperative AKI in unselected cardiac surgery patients and (ii) to assess whether the concentration of the biomarkers reflected severity of AKI.

**Methods:**

In a retrospective observational study, biobank blood plasma samples (n = 924) from patients admitted for elective on-pump cardiac surgery were analysed for suPAR and hsCRP levels. The relation between suPAR and hsCRP-values and AKI (any stage), defined by the KDIGO (Kidney Disease: Improving Global Outcomes) criteria, was assessed using adjusted logistic regression. Further, the association between biomarkers and severity (KDIGO 1, KDIGO 2–3 and renal replacement therapy (RRT)) was assessed using adjusted logistic regression.

**Results:**

Postoperative AKI (any stage) was observed in 327 patients (35.4 %). A doubling of preoperative suPAR corresponded to an adjusted odds ratio (OR) for postoperative AKI (any stage) of 1.62 (95 % CI 1.26–2.09, *p* < 0.001). Furthermore, a doubling of suPAR had an adjusted OR of 1.50 (95 % CI 1.16–1.93, *p* = 0.002), 2.44 (95 % CI 1.56–3.82, *p* < 0.001) and 1.92 (95 % CI 1.15–3.23, *p* = 0.002), for KDIGO 1, KDIGO 2–3 and need for RRT, respectively. No significant association was found between elevated levels of hsCRP and any degree of AKI.

**Conclusions:**

Increasing levels of suPAR, but not hsCRP, were associated with development and severity of AKI following on-pump cardiac surgery.

## Background

Acute kidney injury (AKI) still represents one of the most serious complications following cardiac surgery. Despite advancements in the multidisciplinary management, incidence of AKI following cardiopulmonary bypass (CPB) surgery remains approximately 30 %, but with wide variation in frequency depending of the definitions used and duration of follow-up [1–4]. Moderate to severe AKI is associated with prolonged hospitalisation, higher hospital costs and increased mortality [5, 6]. Furthermore, patients experiencing AKI have more than eightfold risk for subsequent development of chronic kidney disease [7]. CPB surgery causes some degree of ischemia-reperfusion kidney injury in all patients, but the detailed pathophysiology for further transition to AKI is not fully understood [8–10].

Today, serum creatinine continues to be the most commonly used criteria to diagnose AKI; however, it lacks sensitivity and specificity, and the fact that it is lagging behind glomerular filtration rate (GFR) by days compromises its clinical utility. Moreover, cardiac surgery patients tend to be overhydrated due to excessive crystalloid volume required for priming and running the CPB circuit, which may cause underdiagnosing of AKI due to haemodilution [11]. Early and reliable identification of patients at risk is warranted. However, there are challenges in identification of patients at risk using established clinical risk factors, which suggest that some patients may have subclinically impaired renal capacity or predisposition. Therefore, an understanding of the pathophysiological mechanisms and associated cellular pathways is essential in order to identify patients at risk of CPB related AKI and to develop targeted protective interventions.

Since intrarenal and systemic inflammation are recognised as critical components in the development of AKI, identification of biomarkers detecting subclinical levels of inflammation has gained interest [12, 13]. The proinflammatory biomarkers, soluble urokinase-type plasminogen activator receptor (suPAR) and high-sensitivity C-Reactive Protein (hsCRP), reflect different inflammatory pathways [14]. High levels of suPAR have in several studies been associated with a decrease in estimated glomerular filtration rate (eGFR) over time in patients with pre-existing kidney disease [15–17]. Recently, a study found admission suPAR levels was associated with occurrence and severity of AKI in hospitalised COVID-19 patients [18]. Two studies have demonstrated an association between development of acute kidney injury and suPAR levels in cardiac surgery patients [19, 20]. In addition, recent studies point towards that the suPAR protein in experimental models acts synergistically with other kidney-damaging processes, such as ischemia, oxidative stress, cytotoxic drugs, and thereby contributes to development of AKI which is of interest since blockage with antibodies directed to the membrane-bound urokinase-type plasminogen activator receptor (uPAR) in the experimental setting mitigated the kidney injury [20]. Elevated levels of hsCRP are associated with increased frequency of AKI and progression of chronic kidney disease in patients with acute myocardial infarction [21, 22]. Only a few studies have explored the relationship between hsCRP levels and AKI in cardiac surgery patients with divergent findings [23, 24].

Therefore, the aim of this study was to investigate any potential association between postoperative AKI and preoperative elevated levels of suPAR and hsCRP in unselected cardiac surgery patients. Further, to assess whether elevated levels of the biomarkers were associated with severity of AKI.

## Methods

### Study design and population

In a retrospective observational study, biobank blood plasma samples from previous research projects involving adult patients admitted for elective on-pump cardiac surgery, during the period August 2012 to June 2018, at the Department of Cardiothoracic Surgery at Rigshospitalet, Copenhagen University Hospital, were included. Exclusion criteria were peroperatively cancellation of the surgery due to unforeseen anatomic challenges, change to off-pump coronary artery bypass (OPCAB) surgery, death prior to surgery or during the surgical procedure, project plasma samples or preoperative serum creatinine not available and preoperative end-stage kidney disease (defined as receipt of dialysis). The blood plasma samples for analysis of biomarkers were drawn preoperatively or within the first 24 h of surgery (only preoperative hsCRP data were included) and stored at -80^o^C in the PERSIMUNE Biobank or two other biobanks at Department of Cardiothoracic Anaesthesiology and Department of Cardiothoracic Surgery, Rigshospitalet.

### Biochemical analysis

Matrix tubes with 150 µl EDTA-plasma were obtained from biobanks and analysed at the Department of Clinical Biochemistry, Rigshospitalet, by experienced technicians, unaware of clinical data. Measurement of suPAR was performed using suPARnostic® kit (ViroGates), with a lower limit of detection of 0.1 ng per ml, and hsCRP was measured using Tina-quant hsCRP latex assay (Roche/Hitachi), measuring concentrations between 0.3 and 20 mg per l. In patients with CRP > 20 mg per l, a standard CRP-analysis was performed (Tina-quant CRP latex assay (Roche/Hitachi)), measuring concentrations up to 700 mg per l. Both suPAR and hsCRP have previously proven stable in long term frozen storage [25–27]. For suPAR, internal validation revealed an intra-assay coefficient of variance (CV) of 2.3 % based on duplicate measurements in 115 random patients from our cohort. CV was calculated as standard deviation (SD) divided by the duplicate mean and multiplied by 100. CRP and hsCRP were measured by routine analyses at our Hospital laboratory, with a maximum allowed variance (CV_max_) of 4–6 % for control levels 6 and 55 mg per l for CRP and 4–7 % for control levels 0.6 and 7.0 mg per l for hsCRP, respectively.

### Patient characteristics

Information on preoperative comorbidity and anthropometric data was obtained from the admission notes. The most recent echocardiogram was used for assessment of left ventricle ejection fraction (LVEF) and estimation of pulmonary arterial pressure. The local cardiothoracic database (PATS) contained information on admission and discharge, previous percutaneous cardiac interventions (PCI), myocardial infarction within 90 days and previous cardiac surgery. Information on the surgical procedure performed was based on the surgical description from the patients’ records. The local perfusionist database was used to supplement information concerning CPB, including duration and aortic clamp time. All clinical information was attained in collaboration with data processors from PERSIMUNE - Centre of Excellence.

### Measures of kidney function

Serum creatinine closest to surgery (within 30 days) was obtained from the biochemical records and used as baseline level. Creatinine clearance was calculated using the Cockcroft-Gault formula [28], and eGFR was calculated by the Chronic Kidney Disease Epidemiology Collaboration (CKD-EPI) equation [29]. AKI outcomes were calculated as the difference between baseline serum creatinine and the peak postoperative serum creatinine value within 7 days. Primary outcome was AKI (any stage) defined by the KDIGO (Kidney Disease: Improving Global Outcomes) criteria as a postoperative increase in serum creatinine of ≥ 26.5 µmol per l within 48 h of surgery or 1.5 times baseline value [30]. Secondary outcomes were KDIGO stage 1, KDIGO stage 2–3, and need for renal replacement therapy (RRT). KDIGO stage 1 was defined as an increase in serum creatinine levels of 1.5–1.9 times baseline or an absolute increase of ≥ 26.5 µmol per l within 48 h. Stage 2 was defined as an increase in serum creatinine level of 2.0-2.9 times baseline, and stage 3 was 3.0 times or above from baseline levels or initiation of RRT.

### Management of Anaesthesia

All surgical procedures employed CPB with membrane oxygenation and roller pumps with non-pulsatile flow and a normothermic bladder temperature (36.5–37.0 ^o^C). Pump flow was set to 2.4 l per min per m^2^. Arterial blood pressure was monitored with a cannula placed in the left radial artery. Heparinisation was provided to obtain an activated clotting time > 480 s. All patients received triazolam (0.125–0.250 mg) prior to surgery. Anaesthesia was induced with fentanyl (10 µg per kg), propofol (1–2 mg per kg), and cisatracurium (0.1 mg per kg) and maintained with sevoflurane (0.5-3.0 %) and a continuous infusion of remifentanil (15–30 µg per kg per hour). RRT was initiated at the discretion of the treating clinician.

### Statistical analysis

Distribution of continuous variables are described by the median (25th and 75th percentiles) and categorical variables as counts (percentage). Baseline and intraoperative variables were compared using Mann-Whitney U Test for continuous variables and Pearson’s chi-squared test for categorical variables.

Adjusted logistic regression was used to assess any association between AKI development and suPAR and hsCRP values, both as a continuous (log2-transformed) variable, and as empirical quartiles, with the lowest quartile acting as reference to the other quartiles. Adjusted logistic regression was also used to investigate the association between continuous (log2-transformed) values of suPAR and hsCRP, and the secondary outcomes: KDIGO stage 1, KDIGO stage 2–3 and RRT during index hospitalisation. Finally, a subgroup analysis of patients with pre-existing eGFR < 60 ml per minute per 1.73 m^2^ was performed using adjusted logistic regression to investigate the association between AKI development and continuous (log2-transformed) values of suPAR and hsCRP. All models assessing AKI (any stage) as outcome were adjusted for the following known risk factors for AKI: age, sex, diabetes mellitus, arterial hypertension, preoperative ejection fraction, preoperative creatinine and CPB time. Models for the secondary outcomes were adjusted for preoperative eGFR and CPB time > 120 min. Linearity of the continuous variables with respect to the logit of the dependent variable was assessed via the Box-Tidwell (1962) procedure including a Bonferroni correction.

Missing data was handled using pairwise deletion. Statistical analyses were performed using SPSS version 26.0 (IBM SPSS statistics for windows. Armonk, NY, USA) and GraphPad Prism version 8.4.0 (GraphPad software for windows. La Jolla, California, USA). A two-sided *p* value < 0.05 was considered significant.

## Results

**Fig. 1:**
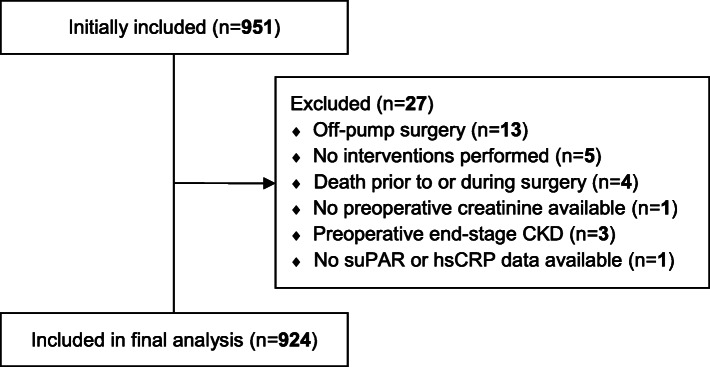
CONSORT diagram

Among 951 patients enrolled in the study, 27 patients were excluded, leaving 924 for final analysis (see CONSORT diagram, Fig. 1). Demographic and clinical characteristics for the entire population and stratified according to occurrence of AKI are presented in Table 1. In short, patients with AKI had a higher EuroSCORE II and more comorbidities, such as diabetes and hypertension. Furthermore, patients developing AKI had higher preoperative serum creatinine levels and correspondingly lower creatinine clearance and eGFR. Also, a prolonged CPB duration and more complex procedures were observed in patients developing AKI.

**Table 1:** – Patient demographics and clinical characteristics

Variable	Total		No AKI		AKI		*p* value
	*n* = 924		*n* = 597		*n* = 327		
**Preoperative data**							
Age	67	(59–73)	67	(59–73)	68	(59–74)	0.08
Male sex	738	(79.9)	470	(78.7)	268	(82.0)	0.27
Body Mass Index (kg m^2 − 1^)	26.8	(24.2–29.9)	26.9	(24.0-29.4)	27.5	(24.8–31.2)	**< 0.001**
Diabetes mellitus							**0.02**
No	740	(80.1)	494	(82.7)	246	(75.2)	-
NIDDM	131	(14.2)	73	(12.2)	58	(17.7)	-
IDDM	53	(5.7)	30	(5.0)	23	(7.0)	-
Arterial hypertension	588	(63.6)	357	(59.8)	231	(70.6)	**0.001**
EuroSCORE II^a^	1.66	(1.01–3.05)	1.46	(0.92–2.54)	2.48	(1.36–4.38)	**< 0.001**
Smoking status							0.45
Never	272	(29.4)	172	(28.8)	100	(30.6)	-
Previous	494	(53.5)	316	(52.9)	178	(54.4)	-
Active	158	(17.1)	109	(18.3)	49	(15.0)	-
Previous PCI	141	(15.3)	94	(15.7)	47	(14.4)	0.63
NYHA class							**0.01**
I	273	(29.5)	194	(32.5)	79	(24.2)	-
II	382	(41.3)	245	(41.0)	137	(41.9)	-
III	232	(2.1)	140	(23.5)	92	(28.1)	-
IV	37	(4.0)	18	(3.0)	19	(5.8)	-
CCS 4^b^	40	(4.6)	28	(4.9)	12	(3.9)	0.51
LVEF	55	(45–60)	60	(45–60)	55	(45–60)	**0.01**
Prev. cardiac surgery	59	(6.4)	25	(4.2)	34	(10.4)	**< 0.001**
Chronic lung disease	81	(8.8)	49	(8.2)	32	(9.8)	0.47
Extracardiac arteriopathy	101	(10.9)	58	(9.7)	43	(13.1)	0.12
Pulmonary hypertension							**0.002**
No	805	(87.1)	537	(89.9)	268	(82.0)	**-**
Moderate (31–55 mmHg)	105	(11.4)	53	(8.9)	52	(15.9)	-
Severe (> 55 mmHg)	14	(1.5)	7	(1.2)	7	(2.1)	-
Urgency	264	(28.6)	183	(30.7)	81	(24.8)	0.07
MI within 90 days	184	(19.9)	113	(18.9)	71	(21.7)	0.34
Baseline serum creatinine (µmol l^− 1^)	85	(74–99)	83	(73–95)	88	(78–106)	**< 0.001**
Creatinine clearance	84	(66–106)	86	(68–107)	79	(62–104)	**0.005**
Estimated glomerular filtration rate (ml min^− 1^ 1.73 m^2 − 1^)	78	(65–90)	81	(68–91)	73	(59–88)	**< 0.001**
hsCRP (mg l^− 1^)^c^	1.9	(1.0-4.5)	1.9	(1.0-4.3)	2.0	(1.0-4.9)	0.16
suPAR (ng ml^− 1^)	2.6	(2.0-3.4)	2.4	(1.9–3.1)	2.9	(2.2–4.1)	**< 0.001**
**Intraoperative data**							
Procedure							**< 0.001**
CABG	458	(49.6)	343	(57.5)	115	(35.2)	-
AVR	140	(15.2)	101	(16.9)	39	(11.9)	-
Other single procedure	58	(6.3)	39	(6.5)	19	(5.8)	-
2 procedures	195	(21.1)	90	(15.1)	105	(32.1)	-
≥ 3 procedures	73	(7.9)	24	(4.0)	49	(15.0)	-
Surgery on thoracic aorta	59	(6.4)	29	(4.9)	30	(9.2)	**0.01**
CPB duration^d^	93	(72–125)	85	(67–106)	120	(87–156)	**< 0.001**
Aorta-clamp-time^e^	60	(43–87)	55	(39–75)	83	(52–114)	**< 0.001**

*NIDDM* Non-insulin‐dependent diabetes mellitus, *IDDM* Insulin‐dependent diabetes mellitus, *NYHA* New York Heart Association functional classification, *CCS* Canadian Cardiovascular Society angina score, *LVEF* left ventricular ejection fraction, *MI* myocardial infarction, *CABG* coronary artery bypass grafting, *AVR* aortic valve replacement, *CPB* Cardiopulmonary bypass. Missing data; (a) 2 cases, (b) 49 cases, (c) 20 cases, (d) 5 cases, (e) 5 cases. Continuous variables are expressed as median (25-75th percentiles) and categorical variables are expressed as count (percentage). *p* values < 0.05 is written in bold.

**Fig. 2:**
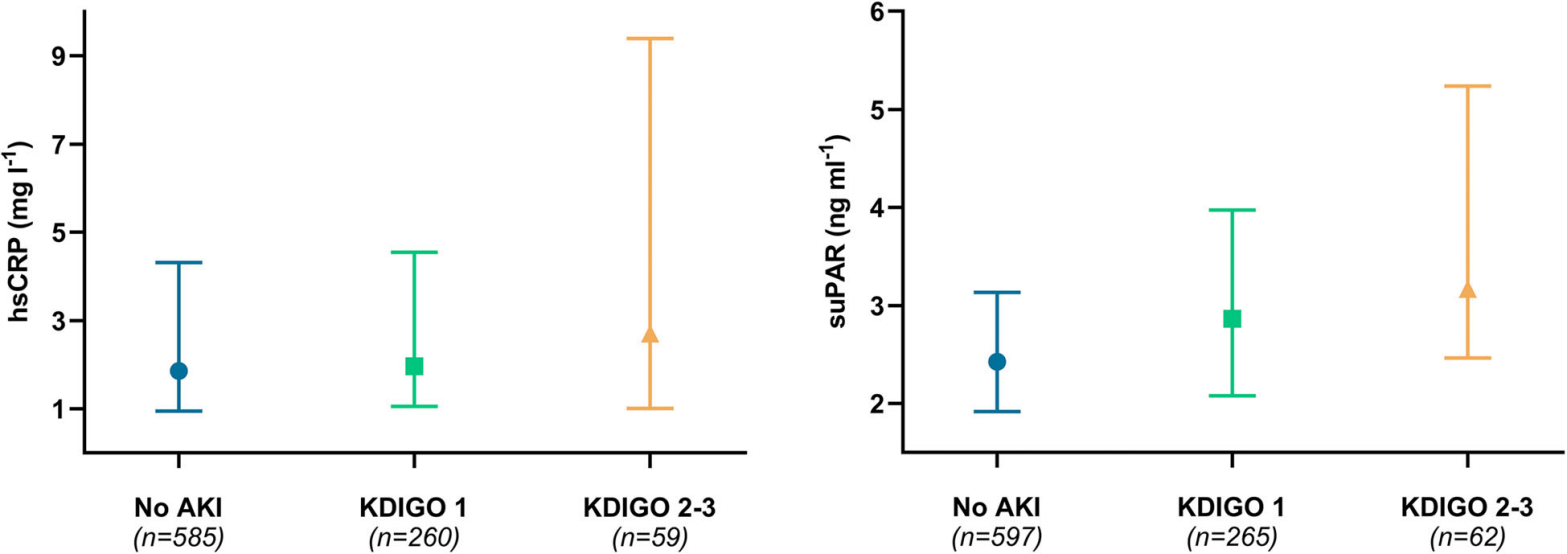
Median and 25-75th percentiles of suPAR and hsCRP in relation to AKI severity

Postoperative AKI was observed in 327 patients (35.4 %), and 26 patients (2.8 %) required RRT during hospitalisation. Severity of AKI was 265 (28.7 %), 33 (3.6 %) and 29 (3.1 %) patients, for KDIGO stage 1, 2 and 3, respectively. Median levels of suPAR and hsCRP increased in accordance with severity of AKI (Fig. 2). Within quartiles of suPAR, the frequency of AKI was 29 %, 25 %, 38 and 50 %, from the 1st to the 4th quartile, respectively. For hsCRP, frequency of AKI was 32 %, 35 %, 36 and 38 %, from the 1st to the 4th quartile, respectively.

**Fig. 3:**
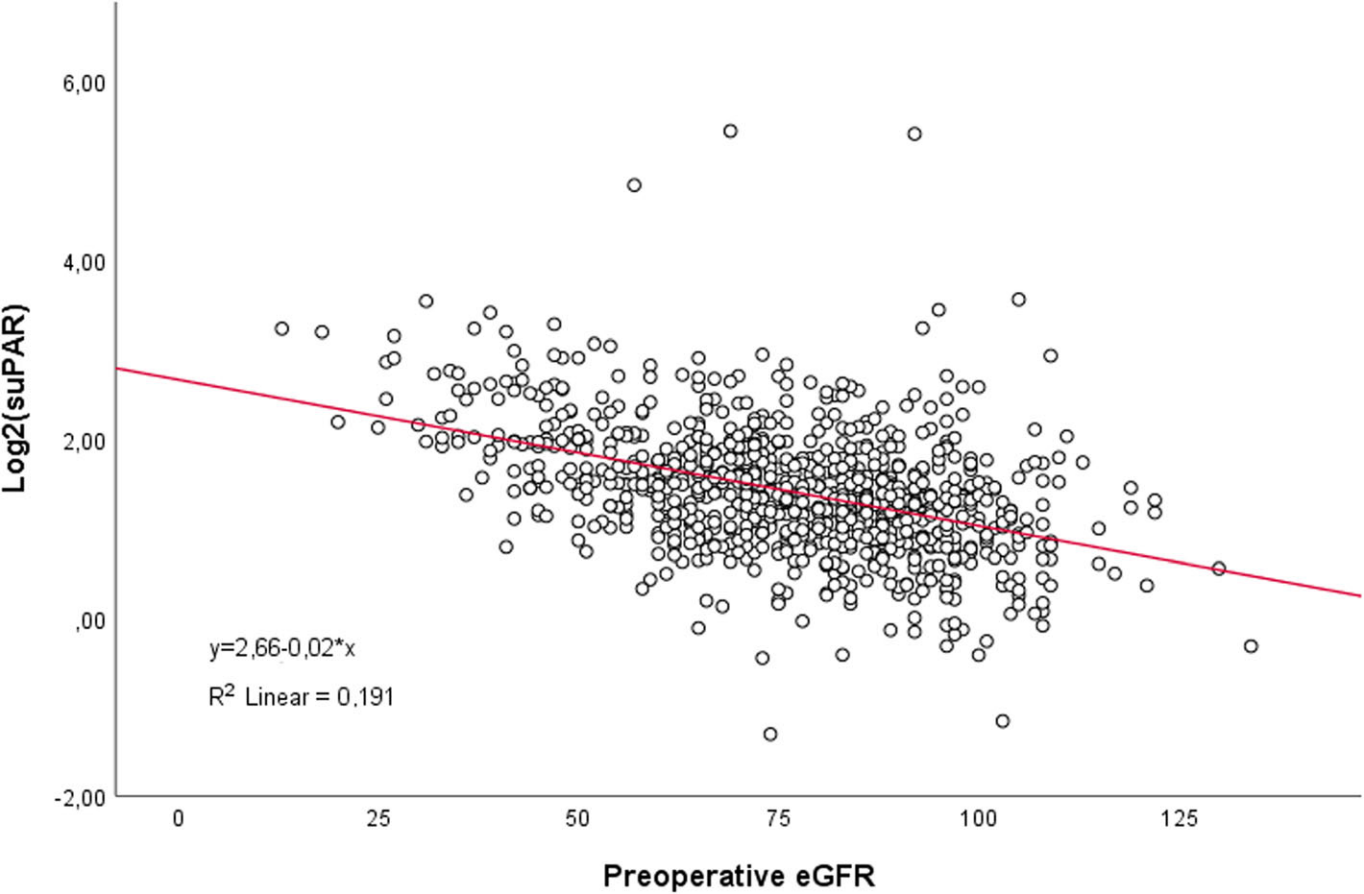
Scatterplot with preoperative eGFR and preoperative log2-transformed suPAR values

Unadjusted logistic regression analysis demonstrated an OR for AKI (any stage) of 1.96 (95% CI 1.59-2.42, *p*<0.001) per doubling of suPAR, whereas the OR for hsCRP was 1.06 (95% CI 0.98-1.15, *p*=0.14) per doubling. Similar ORs were found following adjustment for other risk-factors (Table2). We found a negative correlation between preoperative values of suPAR and eGFR assessed in a linear regression model (Fig. 3). Therefore, to further confirm the findings mentioned above, we performed an additional analysis replacing the covariates; age, gender and preoperative creatinine with preoperative eGFR, and found an OR of 1.59 (95% CI 1.24-2.04, *p*<0.001) per doubling of suPAR. After stratification into individual KDIGO categories, unadjusted analysis revealed an OR per doubling of suPAR of 1.81 (95% CI 1.45-2.27, *p*<0.001), 2.78 (95% CI 1.87-4.13, *p*<0.001) and 2.75 (95% CI 1.78-4.27, *p*<0.001), for KDIGO 1, KDIGO 2-3 and RRT during hospitalisation, respectively. In comparison, a doubling of hsCRP resulted in an OR of 1.04 (95% CI 0.95-1.13, *p*=0.44), 1.19 (1.02-1.38, *p*=0.02), and 1.28 (95% CI 1.03-1.58, *p*=0.03), for KDIGO 1, KDIGO 2-3 and RRT during hospitalisation, respectively. Following adjustment for other risk-factors, only increasing values of suPAR carried significantly higher odds ratios (Table 2).

**Table 2:** – Adjusted logistic regression models – all patients

AKI outcome definitions	suPAR (per doubling)		hsCRP (per doubling)	
Primary outcome	OR (95 % CI)	*p*	OR (95 % CI)	*p*
- AKI (any KDIGO)	1.62 (1.26–2.09)	**< 0.001**	1.05 (0.96–1.15)	0.31
Secondary outcomes				
- KDIGO stage 1	1.50 (1.16–1.93)	**0.002**	1.02 (0.93–1.12)	0.73
- KDIGO stage 2–3	2.44 (1.56–3.82)	**< 0.001**	1.17 (0.99–1.37)	0.06
- RRT during hospitalisation	1.92 (1.15–3.23)	**0.002**	1.17 (0.93–1.48)	0.19

Primary outcome models were adjusted for age, sex, diabetes mellitus, arterial hypertension, preoperative ejection fraction, preoperative creatinine and CPB time. Secondary outcome models were adjusted for preoperative eGFR and CPB time > 120 min. *p* values < 0.05 is written in bold

**Fig. 4:**
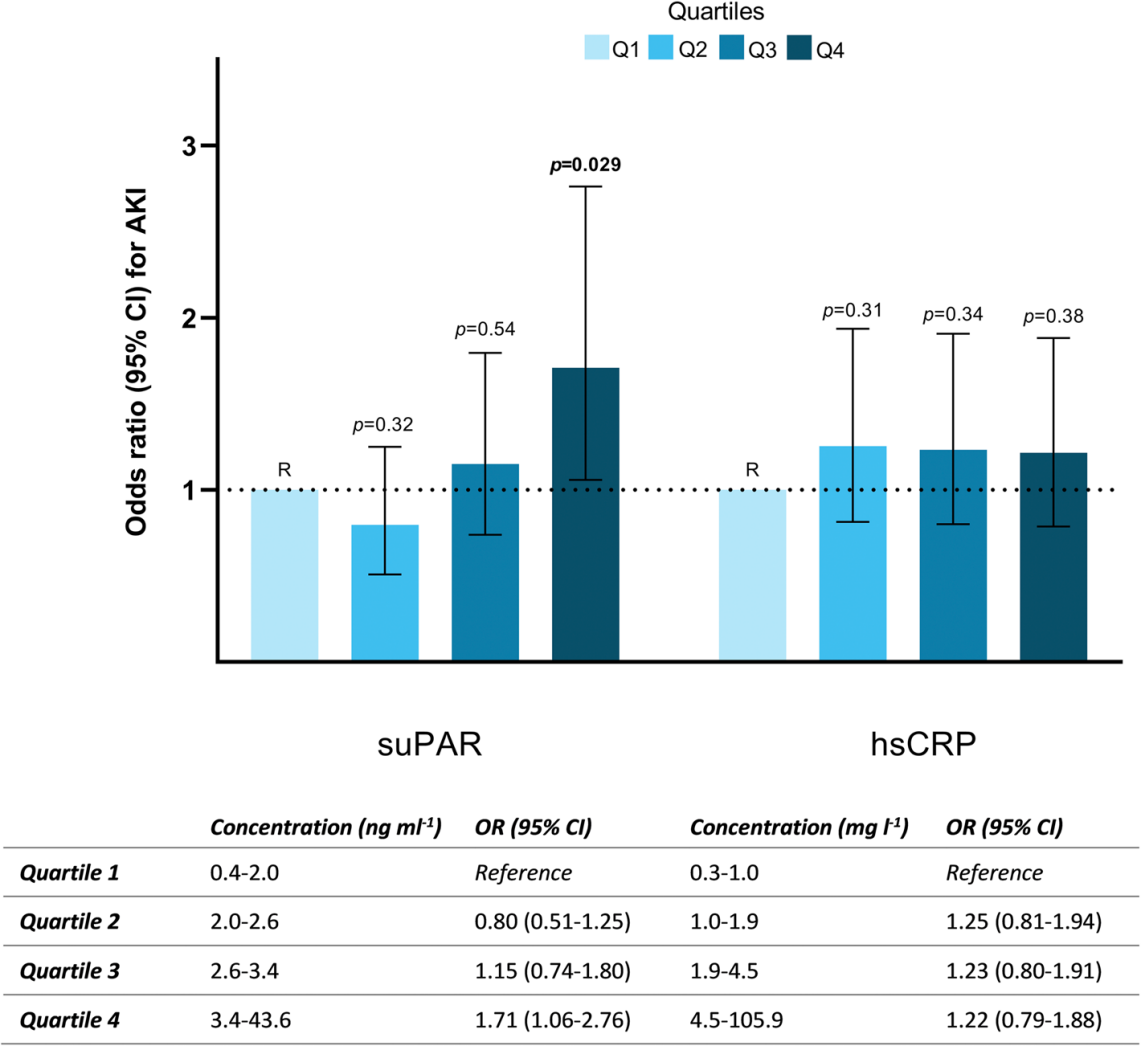
Adjusted odds ratios for AKI development (any stage) according to empirical quartiles of suPAR and hsCRP. Models were adjusted for age, sex, diabetes mellitus, arterial hypertension, preoperative ejection fraction, preoperative creatinine and CPB time. *p* values <0.05 is written in bold.

When assessed as empirical quartiles (1st quartile reference), only the highest quartile of suPAR (> 3.42 ng per ml) carried a significantly increased OR for developing AKI (any stage) of 2.44 (95 % CI 1.67–3.59, *p* < 0.001) in unadjusted analysis, which remained significant following adjustment for other risk factors (Fig. 4). In comparison, increasing quartiles of hsCRP, were not associated with development of AKI (Fig. 4).

In patients with preoperatively reduced renal function, defined as eGFR < 60 ml per minute per 1.73 m^2^, adjusted analysis demonstrated an OR for AKI development (any stage) per doubling of suPAR of 2.88 (95 % CI 1.50–5.55 *p* = 0.002) (*n* = 161), compared to an OR of 1.42 (95 % CI 1.08–1.87, *p* = 0.013), in patients with normal preoperative eGFR (*n* = 763). For hsCRP no association was found irrespective of preoperative eGFR.

**Fig. 5:**
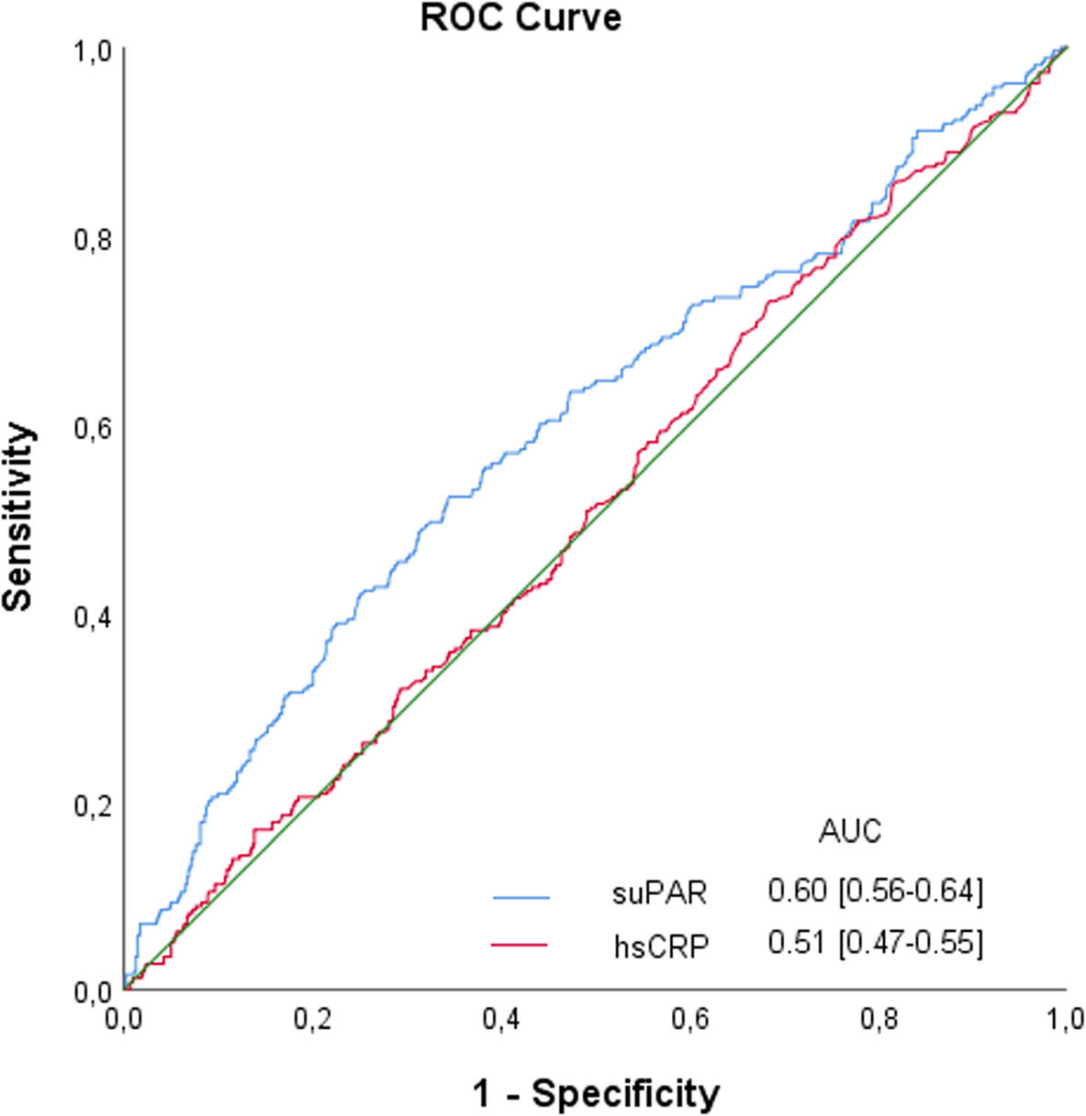
Receiver Operating Characteristic (ROC) curve and AUC for preoperative suPAR and hsCRP values

The ROC curve for preoperative values of hsCRP showed no discriminative ability on postoperative AKI and SuPAR had only limited discriminative ability (Fig. 5), which was comparable to AUC of eGFR 0.57 [0.53-0.61].

## Discussion

In this study including 924 patients we found increasing levels of suPAR were associated with subsequent development of AKI following on-pump cardiac surgery, regardless of baseline kidney function. Further, increasing suPAR-levels were in particular related to more severe stages of AKI. Elevated pre-operative levels of hsCRP were not associated with AKI or any degree thereof.

We found increasing suPAR-levels to be associated with development of AKI, which is consistent with the findings from Hayek and colleagues, demonstrating increasing levels of suPAR to be associated with subsequent AKI development in patients exposed to either radio-opaque contrast material, cardiac surgery or critical illness [20]. However, the cardiac surgery population in the latter study comprised fewer patients (*n* = 250), and two-thirds of the patients had a preoperative eGFR below 60 ml per minute per 1.73 m^2^, which is four times more frequent than in our study. The median suPAR-levels within each quartile were markedly higher in the study by Hayek and colleagues, which together with the preoperative reduced eGFR may explain the differences in risk of AKI between studies [20].

The association between higher levels of suPAR and lower eGFR has previously been described in several studies including patients with pre-existing kidney disease [15–17]. This raises the question, whether suPAR simply reflects eGFR and consequently the increased risk of developing AKI with high suPAR levels is related to pre-existing renal impairment? In a large Swedish study with > 5000 persons from the general population, the suPAR concentration at baseline was not associated with eGFR, but an increased suPAR level predicted a subsequent decrease in eGFR later in life [31]. To explore impact of baseline eGFR, we performed a subgroup analysis among the 161 patients with an eGFR less than 60 ml per minute per 1.73 m^2^ and found that a doubling in suPAR carried almost a four-fold higher odds ratio of AKI compared to patients with normal or mildly impaired renal function. Nevertheless, increasing levels of suPAR remained a significant predictor of postoperative AKI also among patients without renal impairment, in line with previous findings [19].

The preoperative concentration of hsCRP was not associated with AKI development following adjustment for other known risk factors. These results are in contrast to Han and colleagues observations, where increasing hsCRP-levels were associated with postoperative AKI in patients undergoing exclusively CABG-procedures [23]. Han and colleagues used the same KDIGO criteria, and found comparable AKI occurrence as in our study, but 25 % of the patients were emergency procedures, which is known to carry an increased risk of AKI, partly due to a shorter time interval between coronary angiography and the CABG procedure [32]. Another Scandinavian study evaluated the prognostic ability of multiple biomarkers, including hsCRP, for postoperative AKI, in a comparable cardiac surgery population, and in agreement with the present study a high hsCRP was not associated with an increased risk of AKI [24].

Although the pathophysiological mechanisms of suPAR and hsCRP in kidney injury are not fully understood, some causality has been proposed. Exposure to suPAR upregulates and activates αvβ3 integrin expressed in podocytes, which ultimately causes cell detachment and proteinuria [33, 34]. Recently, suPAR has also demonstrated to increase the cellular energy demands and inflict oxidative stress in tubular cells, which is considered particularly susceptible to ischemia-reperfusion injury [20]. Since a higher level of suPAR may also be a result of increased uPAR activity, several studies have investigated uPAR and found it essential for the activation of αvβ3 integrin and hence a cause to proteinuria via effacement of podocyte foot processes [35]. Further, a possible mechanism of kidney injury is by a complex formation of plasminogen activator inhibitor-1 (PAI-1), urokinase-type plasminogen activator (uPA), and uPAR on the podocyte surface, which further binds β1 integrin and causes podocyte detachment [36]. In summary, it seems clear that suPAR (and uPAR) has an important role in remodelling the filtration barrier, but also affects tubular epithelial cells during kidney injury. As already mentioned, a recent study demonstrated that treatment with monoclonal antibodies targeting uPAR could attenuate kidney injury in transgenic mice overexpressing suPAR [20]. Thus, modulation of suPAR and its associated pathways could be a potential target in patients with elevated plasma suPAR levels.

Since many of the cellular mediators and triggers are both responsible for the initial parenchymal damage and subsequent repair and regeneration in AKI, studies investigating interventional strategies are warranted, but must be performed with caution in terms of timing and target [12]. Today, most experimental research models are performed in rodents, although these animals have quite different physiology compared to humans. Future studies should include experimental models of larger mammals, such as pigs, with a closer resemblance to the human immune system and anatomy [37, 38].

Several strengths exist in this present study, including the comprehensive data material with few missing data, the parallel analysis of the two biomarkers and the fact that we were able to accurately assess the frequency and severity of AKI within a 7-day postoperative period. However, some important limitations must be considered, too. The retrospective nature of the study only provides associations, not causality, thereby only allowing hypothesis generation. The blood samples were obtained in relations to previous research projects on patients undergoing cardiac surgery, which may introduce selection bias. Observations are based on a single cardiac centre with mainly Caucasian patients, which limits generalisability to other centres and ethnicities. The fact that biomarkers were measured in biobank samples, also represents some uncertainty. However, both suPAR and hsCRP have previously proven stable during long-term frozen storage [25–27]. We also included blood plasma samples for suPAR measurements in patients having only postoperative blood samples available (2 %), since suPAR have previously proven stable for 24 h in patients with ST-elevation myocardial infarction undergoing PCI as well as in patients having CABG procedures involving CPB, contrary to CRP levels [39–41].

## Conclusions

In conclusion, we found that elevated levels of suPAR, but not hsCRP, were associated with development and severity of AKI following on-pump cardiac surgery. Future experimental studies are warranted in order to further investigate the role of suPAR in the underlying pathophysiology of AKI with the potential to develop a targeted intervention.

## Data Availability

The datasets used and analysed during the current study are available from the corresponding author on reasonable request.

## Abbreviations

AKI: Acute kidney injury
suPAR: Soluble urokinase-type plasminogen activator receptor
hsCRP: High-sensitivity C-Reactive Protein
KDIGO: Kidney Disease:Improving Global Outcomes
RRT: Renal replacement therapy
OR: Odds ratio
CI: Confidence interval
CPB: Cardiopulmonary bypass
GFR: Glomerular filtration rate
eGFR: Estimated glomerular filtration rate
uPAR: Urokinase-type plasminogen activator receptor
OPCAB: Off-pump coronary artery bypass
PERSIMUNE: Centre Of Excellence For Personalized Medicine Of Infectious Complications In Immune Deficiency, Rigshospitalet, Denmark
EDTA: Ethylenediamine tetraacetic acid
CV: Coefficient of variance
SD: Standard deviation
LVEF: Left ventricle ejection fraction
PCI: Percutaneous cardiac interventions
EuroSCORE II: The European System for Cardiac Operative Risk Evaluation II
CABG: Coronary artery bypass grafting
PAI-1: Plasminogen activator inhibitor-1
uPA: Urokinase-type plasminogen activator
